# The landscape of small cell lung cancer metastases: Organ specificity and timing

**DOI:** 10.1111/1759-7714.13854

**Published:** 2021-02-03

**Authors:** Zsolt Megyesfalvi, Bernadett Tallosy, Orsolya Pipek, Janos Fillinger, Christian Lang, Thomas Klikovits, Anna Schwendenwein, Mir Alireza Hoda, Ferenc Renyi‐Vamos, Viktoria Laszlo, Melinda Rezeli, Judit Moldvay, Balazs Dome

**Affiliations:** ^1^ Department of Thoracic Surgery Semmelweis University and National Institute of Oncology Budapest Hungary; ^2^ National Koranyi Institute of Pulmonology Budapest Hungary; ^3^ Department of Thoracic Surgery, Comprehensive Cancer Center Medical University of Vienna Vienna Austria; ^4^ Department of Physics of Complex Systems Eötvös Loránd University Budapest Hungary; ^5^ Department of Biomedical Engineering Lund University Lund Sweden; ^6^ 2nd Department of Pathology Semmelweis University Budapest Hungary; ^7^ MTA‐SE NAP, Brain Metastasis Research Group Hungarian Academy of Sciences Budapest Hungary

**Keywords:** metastasis pattern, small cell lung cancer, survival

## Abstract

**Background:**

Early metastasis is a hallmark of small cell lung cancer (SCLC). However, the mechanisms and resulting patterns of SCLC dissemination are unclear. Our aim was thus to investigate the organ specificity and timing of blood‐borne metastases in a comprehensive large cohort of SCLC patients.

**Methods:**

In this retrospective non‐interventional cross‐sectional study of 1009 Caucasian SCLC patients, we investigated the correlation between the distinct locations of the primary tumor and metastatic sites.

**Results:**

The onset of bone (*p* < 0.001), brain (*p* < 0.001), and pericardial (*p* = 0.02) metastases were late events, whereas adrenal gland (*p* = 0.005) and liver (*p* < 0.001) metastases occurred earlier. No significant difference was found in the distribution of early versus late metastases when comparing central and peripheral primary tumors. Patients with bone metastases had a higher than expected likelihood of having liver metastases, while brain metastases tended to appear together with adrenal gland metastases. Pleural and both lung and pericardial metastases also tended to co‐metastasize together more frequently than expected if metastatic events occurred independently. Notably, patients with central primary tumors had decreased median overall survival (OS) compared to those with peripheral tumors, although this tendency does not appear to be significant (*p* = 0.072).

**Conclusion:**

Our results are suggestive for particular site‐ and sequence‐specific metastasis patterns in human SCLC. SCLC bone metastases tend to appear together with liver metastases, while brain metastases occur together with adrenal gland metastases. Better understanding of metastasis distribution patterns might help to improve the diagnosis and therapeutic decision‐making in SCLC patients.

## Graphical abstract

In this study, our aim was to examine the organ specificity and timing of blood‐borne metastases in a comprehensive large cohort of Caucasian SCLC patients (*n*

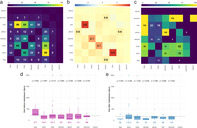

=1009). We report here that the onset of bone, brain, and pericardial metastases are late events, whereas adrenal gland and liver metastases occur earlier during tumor progression. We also show that several metastatic sites, such as bone and liver, and brain and adrenal gland intend to co‐metastasize preferentially. Of note, however, no significant associations were observed with regards to the timing and organ preference of distant metastases when comparing central and peripheral primary tumors.

## INTRODUCTION

Small cell lung cancer (SCLC; comprising approximately 15% of all lung cancers and having a five‐year survival of 7%) is an aggressive tumor characterized by rapid growth, genomic instability, and the development of early metastases.^1^, ^2^ Because widespread metastases and exceptional metastatic potential are a major part of SCLC behaviour, in the majority of cases SCLC has already metastasized to sites outside the chest at the time of diagnosis.^3^, ^4^ The most common sites of metastasis include the brain, bones, liver, and adrenal glands.^5^ Regardless of the location of the metastatic site, patients with extrathoracic metastases have markedly shortened survival compared to those without distant organ metastases at diagnosis.^6^ Furthermore, depending on the involved organ, distant metastases are also associated with pain, neurological disorders, and impaired quality of life.^7^, ^8^ However, conflicting data exist regarding the pattern, timing, and co‐occurrence of SCLC metastases. Our group previously found that in lung adenocarcinoma patients bone metastases tend to appear together with adrenal gland and liver metastases, while pleural and pericardial, and skin and adrenal gland metastasis pairs also appear more frequently together than expected if metastatic events occurred independently.^9^ Of note, a very recent large population‐based study on unselected Asian lung cancer patients also suggested that bone metastases tended to onset together with liver metastases.^10^ To date, however, no metastasis pattern‐specific analyses have been conducted in Caucasian SCLC patients.

The clinical armamentarium and therapeutic approaches for patients with metastatic SCLC have changed only minimally over the past 30 years.^2^, ^4^, ^11^ Accordingly, the standard‐of‐care chemotherapy (CHT) regimen for these patients still consists of a platinum agent (cisplatin or carboplatin) combined with etoposide, with or without thoracic irradiation.^11^, ^12^ Unlike non‐SCLC (NSCLC), which has an intrinsic tendency for CHT resistance, SCLC is tantalizingly chemosensitive and up to 75% of all cases initially respond to platinum‐based regimens.^2^, ^13^ Yet, despite the high initial response rates, most patients with SCLC experience relapse within 2 years and die from systemic metastasis.^14^ Furthermore, certain types of extrathoracic metastases might also influence the response rates. Specifically, both liver and brain metastases correlate with an unfavourable response to platinum‐based CHT.^5^ In addition, patients with distant organ metastases frequently require other therapeutic interventions as well, including bisphosphonate therapy or palliative radiotherapy in case of bone metastases and whole brain radiation therapy (WBRT) in case of brain metastases.^15^, ^16^, ^17^

Importantly, however, although distant metastases have a significant impact on therapeutic approaches and are considered a major factor for unfavourable prognosis, metastatic patterns and their influence on survival have not been extensively analysed in SCLC. For this reason, the aim of our cross‐sectional study was to examine the organ specificity and timing of blood‐borne metastases in a comprehensive large cohort of Caucasian SCLC patients.

## PATIENTS AND METHODS

### Study population

In this single‐center non‐interventional study, we included 1009 SCLC patients receiving standard‐of‐care therapy between 1999 and 2019 at the National Koranyi Institute of Pulmonology, Budapest, Hungary (Table 1). Patients were diagnosed either cytologically or histologically, and all patients underwent bronchoscopical examination. Based on the study aims, predefined data were collected retrospectively, focusing on the bronchoscopic localization of the primary tumor and distant metastases at diagnosis and during disease progression. Patients with all stages were included. After primary data collection, the medical records of all patients were systematically reviewed case by case by an independent clinician to ensure completeness and correctness of data. Clinical data regarding age at the time of diagnosis (continuous, interval), gender (dichotomous), smoking status (nominal and dichotomized for the multivariate Cox regression analysis), clinical stage according to the 7th edition of the TNM staging system (dichotomous), location and date of metastases (dichotomous, date), number of metastatic sites (continuous, interval), and survival data (continuous, interval) for the included patients were retrospectively collected from the medical records and/or records from the National Health Insurance Office or Central Statistical Office. With regards to the localization of the primary tumor, endoscopically visible primary SCLCs were defined as central, otherwise as peripheral tumors. Meanwhile, the presence of distant organ metastases was diagnosed by computed tomography (CT) scan, magnetic resonance imaging (MRI), PET‐CT scan, aspiration cytology (e.g. in case of skin metastases), and clinical examination. Metastases that were identified at the time of diagnosis or appeared no later than 30 days after the initial diagnosis were considered to be early, otherwise as late metastases. All therapeutic approaches were conducted based on the individual institutional guidelines in accordance with the current National Comprehensive Cancer Network (NCCN) guidelines.^11^ With regards to CHT agents, patients were treated either with a platinum‐etoposide doublet regimen or with a combination of cyclophosphamide, epirubicin, and vincristine (CEV). Overall survival (OS) was estimated from the time of diagnosis until death of any cause or the last available follow‐up visit. Clinical follow‐up was closed on 1 November 2019.

**TABLE 1:** General clinicopathological characteristics, tumor location, and metastatic spread in SCLC patients

		Total	Median OS (days)	Single organ	Multiple organ
All patients		1009	336	372 (37%)	281 (28%)
Gender	Male	568 (56%)	332	217 (58%)	161 (57%)
	Female	441 (44%)	343	155 (42%)	120 (43%)
Smoking history	Never	37 (4%)	336	12 (3%)	8 (3%)
	Ex	161 (16%)	299	66 (18%)	39 (14%)
	Current	552 (55%)	351	190 (51%)	170 (60%)
	N/A	259 (25%)	321	104 (28%)	64 (23%)
Localization of the primary tumor	Central	830 (82%)	321	315 (85%)	229 (81%)
	Peripheral	148 (15%)	398	50 (13%)	45 (16%)
	N/A	31 (3%)	475	7 (2%)	7 (3%)
Stage at diagnosis	I	1 (<1%)	N/A	1 (<1%)	0 (0%)
	II	4 (<1%)	N/A	1 (<1%)	0 (0%)
	III	30 (3%)	463	13 (3%)	6 (2%)
	IV	42 (4%)	277	25 (7%)	17 (6%)
	N/A	932 (92%)	333	332 (89%)	258 (92%)
Median OS (days)				349	333

*Note*: Data shown in parentheses are column percentages. OS, overall survival; N/A, not available.

### Ethics statement

The present study was performed in accordance with the guidelines of the Helsinki Declaration of the World Medical Association. The national level ethics committee (Hungarian Scientific and Research Ethics Committee of the Medical Research Council, ETT‐TUKEB 23636–2/2018, 23 636/10/2018/EÜIG) approved the study. Due to its retrospective nature, the requirement for written informed consent was waived. After clinical information was collected, patient identifiers were removed and subsequently patients could not be identified either directly or indirectly.

### Statistical analyses

All statistical analyses were performed using R version 3.6.3 (R Foundation for Statistical Computing, Vienna, Austria). Categorical and ordinal parameters including the localization of the primary tumor and the organ‐specific metastatic patterns were statistically analysed by *χ* ^2^ test or Fisher's exact test. Survival curves were estimated by Kaplan–Meier plots and the differences between the groups were compared using the log‐rank test. Median follow‐up time was estimated using the reverse‐censored Kaplan–Meier method. The independent prognostic value of the clinicopathological variables was studied with the Cox proportional hazard regression model. Order preferences and joint probabilities of metastases were investigated as described previously.^9^ Mean interarrival times between different metastatic sites were compared with the Wilcoxon test with the null hypothesis that the mean elapsed time before/after the appearance of a metastasis in the given organ is not different from the mean elapsed time before/after the appearance of a metastasis in any organ. *p* values of less than 0.05 were considered statistically significant and in case of multiple comparisons Bonferroni correction was used.

## RESULTS

### Patients characteristics and metastatic sites

A total of 1009 SCLC patients was included in this study. The median age was 63 years (range 30–91). Patients were predominantly male (56%) and all had Caucasian backgrounds (Table 1). We identified 372 patients with single‐organ metastatic disease and 281 with metastases affecting multiple organs (Table 1). Of these 350 patients already had early metastases, whereas another 285 patients developed late metastases (Table 2). As for the localization of metastases, the most frequent metastatic sites were the liver (*n* = 335), the brain (*n* = 266), the bones (*n* = 192), and the adrenal gland (*n* = 104), followed by pleural (*n* = 55), lung (*n* = 53), pericardial (*n* = 26), and skin (*n* = 4) metastases (Table 2).

**TABLE 2:** Clinicopathological characteristics of different metastatic sites in SCLC patients

Metastatic site^a^		Total	Lung	Bone	Brain	Adrenal	Pleura	Liver	Pericard.	Skin
All patients		1009	53	192	266	104	55	335	26	4
Gender	Male	568 (56%)	30 (57%)	102 (53%)	146 (55%)	70 (67%)	35 (64%)	203 (61%)	15 (58%)	1 (25%)
	Female	441 (44%)	23 (43%)	90 (47%)	120 (45%)	34 (33%)	20 (36%)	123 (39%)	11 (42%)	3 (75%)
Smoking	Never	37 (4%)	1 (2%)	11 (6%)	6 (2%)	1 (1%)	1 (2%)	11 (3%)	3 (12%)	0 (0%)
	Former	161 (16%)	11 (21%)	25 (13%)	42 (16%)	14 (13%)	16 (29%)	49 (15%)	6 (23%)	0 (0%)
	Current	552 (55%)	33 (62%)	105 (55%)	152 (57%)	68 (66%)	27 (49%)	189 (56%)	12 (46%)	2 (50%)
	N/A	259 (25%)	8 (15%)	51 (26%)	66 (25%)	21 (20%)	11 (20%)	86 (26%)	5 (19%)	2 (50%)
Primary tumor location	Central	830 (82%)	39 (74%)	157 (82%)	226 (85%)	83 (80%)	45 (82%)	284 (85%)	20 (77%)	4 (100%)
	Peripheral	148 (15%)	10 (19%)	30 (16%)	35 (13%)	20 (19%)	8 (15%)	47 (14%)	4 (15%)	0 (0%)
	N/A	31 (3%)	4 (7%)	5 (2%)	5 (2%)	1 (1%)	2 (3%)	4 (1%)	2 (8%)	0 (0%)
Stage at diagnosis	I	1 (<1%)	0 (0%)	0 (0%)	1 (<1%)	0 (0%)	0 (0%)	0 (0%)	0 (0%)	0 (0%)
	II	4 (<1%)	0 (0%)	0 (0%)	0 (0%)	0 (0%)	0 (0%)	1 (<1%)	0 (0%)	0 (0%)
	III	30 (3%)	2 (4%)	4 (2%)	10 (4%)	2 (2%)	4 (7%)	6 (2%)	0 (0%)	0 (0%)
	IV	42 (4%)	7 (13%)	13 (7%)	14 (5%)	8 (8%)	2 (4%)	20 (6%)	0 (0%)	0 (0%)
	N/A	932 (92%)	44 (83%)	175 (91%)	241 (91%)	94 (90%)	49 (89%)	308 (92%)	26 (100%)	4 (100%)
Appearance of mets	early	350 (35%)	33 (62%)	59 (31%)	51 (19%)	64 (62%)	22 (40%)	209 (62%)	6 (23%)	1 (25%)
	late	285 (28%)	17 (32%)	131 (68%)	206 (78%)	32 (31%)	33 (60%)	109 (33%)	20 (77%)	2 (50%)
	N/A	18 (2%)	3 (6%)	2 (1%)	9 (3%)	8 (7%)	0 (0%)	17 (5%)	0 (0%)	1 (25%)
Organ distribution of early mets^b^	single organ	275 (79%)	23 (70%)	25 (42%)	29 (57%)	32 (50%)	11 (50%)	151 (72%)	3 (50%)	1 (100%)
	multiple organ	75 (21%)	10 (30%)	34 (58%)	22 (43%)	32 (50%)	11 (50%)	58 (28%)	3 (50%)	0 (0%)
Median OS (days) single organ early mets^b^		254	473	212	225	301	228	238	238	N/A
Median OS (days) multiple organ early mets^b^		175	45	183	71	183	137	183	100	N/A

*Note*: Data shown in parentheses are column percentages. mets, metastases; N/A, not available; OS, overall survival; pericard., pericardial.

a Includes single and multiple organ metastatic disease.

b At the time of diagnosis.

### Pattern, timing, and co‐occurrence of SCLC metastases

**FIGURE 1:**
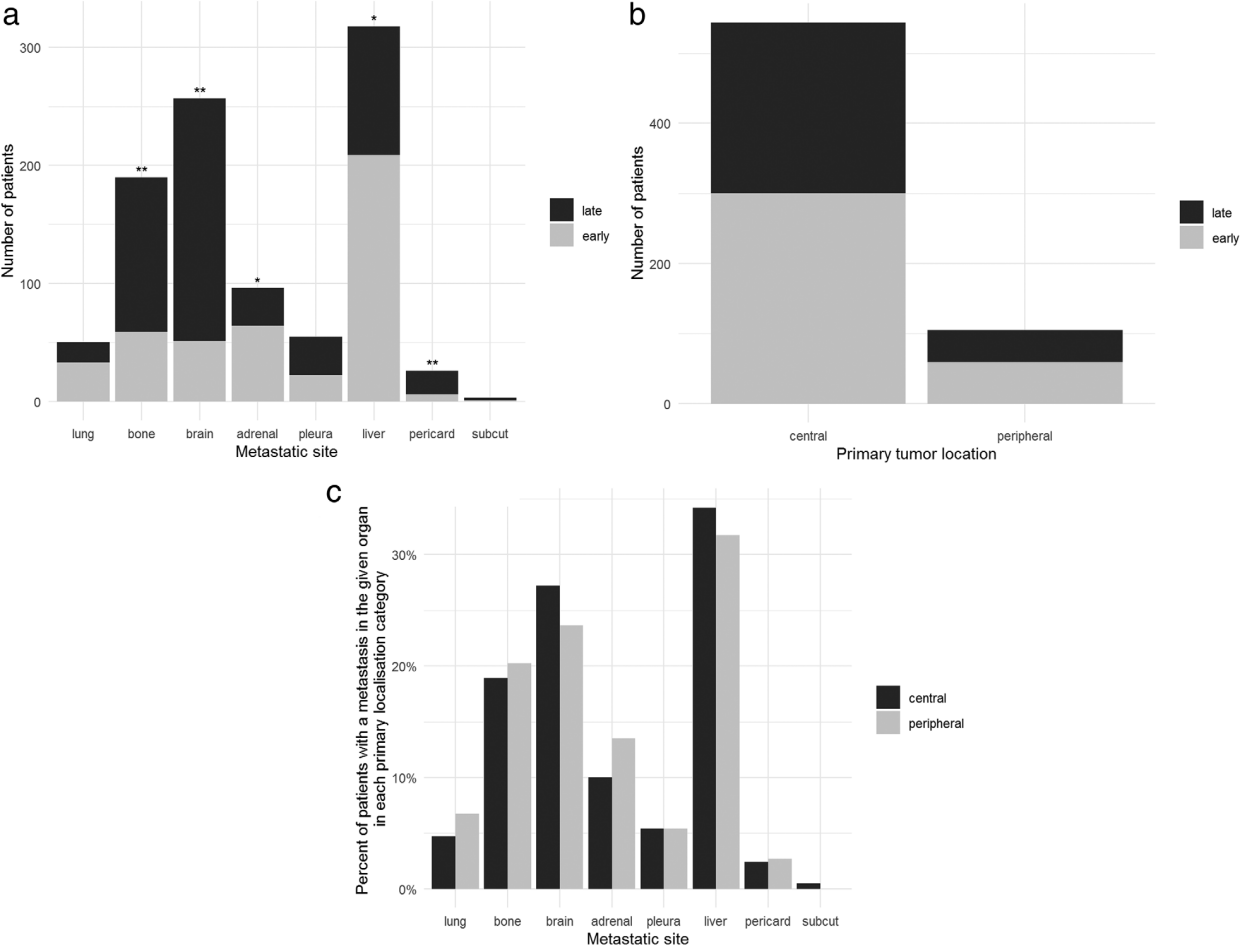
Metastatic site‐specific time sequence in 1009 SCLC patients. (a) The onset of bone (**p < 0.001), brain (**p < 0.001), and pericardial (**p = 0.02) metastases tended to be late, whereas the development of adrenal gland (p* = 0.005) and liver (*p < 0.001) metastases were usually early events during SCLC progression (*favors early metastases, **favors late metastases, Chi‐square test with Bonferroni‐correction). (b) There was no statistically significant difference in the distribution of early vs. late metastasis appearance when comparing centrally and peripherally located primary SCLCs. (c) The percentage of SCLC patients with metastases in each organ according to the corresponding primary tumor's bronchoscopic localization (central or peripheral)

Investigating the metastatic site‐specific time sequence, we found that the onset of bone (*p* < 0.001), brain (*p* < 0.001), and pericardial (*p* = 0.02) metastases tended to be late, whereas the development of adrenal gland (*p* = 0.005) and liver (*p* < 0.001) metastases were usually early events during tumor progression in SCLC patients. No similar observations were obtained for lung, pleural or skin metastases (Figure 1(a) and Table 2). Significantly more patients had central primary tumors (vs. peripheral, *p* < 0.001; Table 1), but no significant association between the localization of primary tumors and metastatic timing was observed (Figure 1(b)). Also, the localization of the primary tumors had no impact on the organ specificity of SCLC metastases (Figure 1(c)).

**FIGURE 2:**
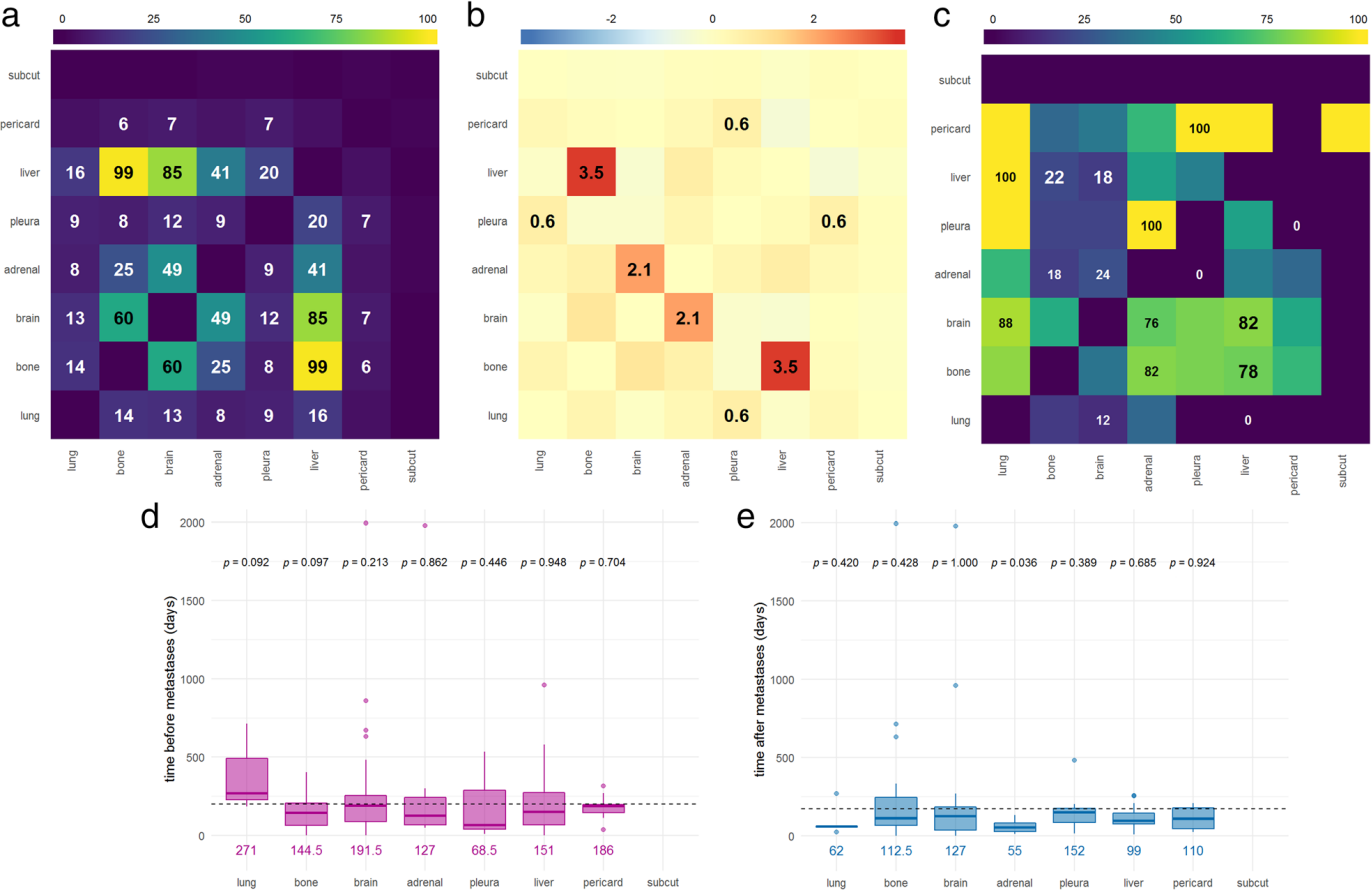
Metastatic patterns and interarrival times before and after the appearance of SCLC metastases in specific organs. (a) The number in the boxes represents the total number of patients with metastases in both organs *i* (*x* axis) and *j* (*y* axis). Based on a gradient scale from blue to yellow, combinations with weak association are highlighted in blue, whereas those with strong association are highlighted in yellow. The number of co‐occurrences is not displayed for organ pairs where the number of observed cases was no larger than five. The total number of patients is 1009, but given that a single patient does not necessarily have metastases in exactly two organs, the boxes do not add up to this. (b) Differences (in percentages) between the estimated joint probability of a patient having metastases in both corresponding organs (*i* and *j*) observed from the actual data and the theoretical values, assuming that metastases appear independently. Metastasis pairs with Arabic numerals indicate that the results remained significant with Bonferroni correction. The rest of the results are nonsignificant or became nonsignificant under multiple testing correction. Lower than expected vs. higher than expected probabilities are highlighted in blue and red, respectively. (c) Order preference of metastatic sites, calculated as the ratio of cases with a metastasis sooner in organ *i* (*x* axis) than in *j* (*y* axis) out of all cases with metastases in both organs (cases where both metastases were detected at diagnosis or where the date of metastasis appearance is ambiguous were excluded). More common orders are highlighted with yellow boxes, while rare ones with blue. (d) Interarrival times before the appearance of metastases in specific organs. Colored numbers below the box plots display the median results. *p* values at the upper part of the figure indicate the results of testing (Wilcoxon test) the null hypothesis that the mean elapsed time before the appearance of a metastasis in the given organ is no different from the mean elapsed time before the appearance of a metastasis in any organ. The horizontal dashed line represents the latter value. Early metastases were excluded from the analysis due to the ambiguity in their onset times. (e) Interarrival times after the appearance of metastases in specific organs. *p* values at the upper part of the figure indicate the results of testing (Wilcoxon test) the null hypothesis that the mean elapsed time after the appearance of a metastasis in the given organ is no different from the mean elapsed time after the appearance of a metastasis in any organ

Next, to examine which metastases are likely to onset together in SCLC patients, we determined the number of cases with every possible metastatic pairs. We found that bone metastases tended to appear together with liver (*N*_*ij*_ = 99) and brain (*N*_*ij*_ = 60) metastases. Moreover, brain and liver metastasis pairs were also fairly common (*N*_*ij*_ = 85; Figure 2(a)).

When analysing whether the estimated joint probability of a patient having metastases in both organs *i* and *j* differed significantly from the theoretical value based on independent incidences, we found that patients with bone metastases had a higher than expected likelihood of also having liver metastases (*d*_*ij*_ = 3.5%) (Figure 2(b)). Brain and adrenal gland metastasis pairs were also more common (*d*_*ij*_ = 2.1%) than suggested by independence (Figure 2(b)). As for intrathoracic metastases, pleural and both lung (*d*_*ij*_ = 0.6%) and pericardial (*d*_*ij*_ = 0.6%) metastases appeared slightly more frequently together than expected. Of note, these results all remain significant at a 0.05 significance level with the use of Bonferroni correction.

To investigate whether there are metastasis pairs where one of the metastases usually tends to appear sooner than the other one given that they both appear together in a single patient, we evaluated the order preference of metastatic sites, as shown in Figure 2(c). Thus, we found that liver metastases usually precede bone (78% of the cases) and brain (82% of the cases) metastases. These results also remained significant even after multiple testing correction. In addition, we also found that lung metastases preceded pleural, pericardial, and liver metastases in all cases, and brain metastases in 88% of the cases, but these results did not remain significant after Bonferroni correction. Similarly, adrenal gland metastases tended to appear sooner than pleural, brain, and bone metastases in 100%, 76%, and 86% of the cases, respectively. However, these results were also nonsignificant. Because the time of appearance of a certain type of metastasis can have both prognostic‐ and treatment‐related relevance, we evaluated the interarrival times before and after the appearance of metastases in specific organs. As shown in Figure 2(d), none of the organs had a statistically significant tendency to present a metastasis sooner or later than other organs when analysing the timing of late metastases. Nevertheless, as for the interarrival times after the appearance of metastases in specific organs, we found that the time that passes after an adrenal gland metastasis before the appearance of another one is shorter (*p* = 0.036) than the average (Figure 2(e)). This result, however, does not remain significant after Bonferroni correction.

### Survival outcomes are influenced by the number of metastatic sites

**FIGURE 3:**
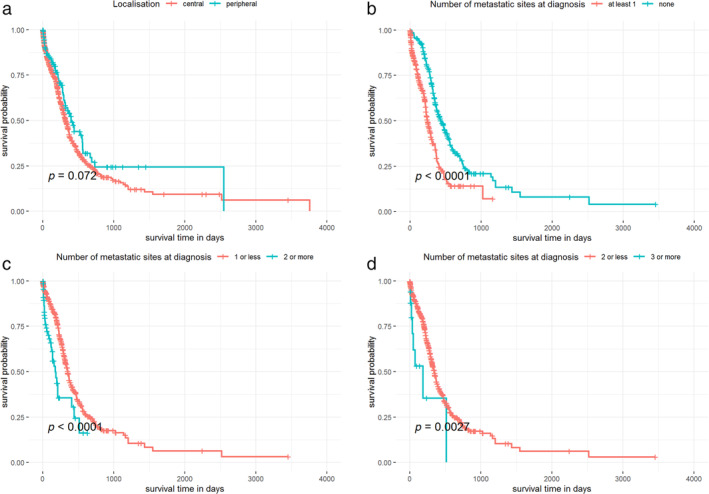
Kaplan–Meier plots for overall survival (OS) in SCLC patients with regards to the primary tumors' localization and the number of metastatic sites. (a) Patients with centrally located primary tumors have decreased survival outcomes compared to those with peripheral tumors, although this tendency did not appear to be statistically significant (*p* = 0.072). (b–d) Patients with at least one metastatic site at diagnosis exhibited worse median OS than those without metastasis (log‐rank *p* < 0.0001). Patients with multiple metastases have worse OS than those with no or a single metastasis at diagnosis (log‐rank *p* < 0.0001). Patients with three or more metastases at diagnosis have lower survival rates than those with at most two metastases (log‐rank *p* = 0.0027)

The median follow‐up time for the total cohort of 1009 patients was 12.6 months. Median survival of the entire group was 11.0 months (95% confidence interval [CI] 9.99–11.97). With regards to the localization of the primary tumor, although patients with centrally located primary tumors have visibly worse survival outcomes compared to those with peripheral SCLC, this tendency does not appear to be statistically significant (median OS, 321 vs. 398 days, respectively, *p* = 0.072; Figure 3(a)). Next, we compared the number of metastatic sites with survival outcomes (Figures 3(b)–(d)) and found that patients with at least one metastatic site at diagnosis exhibit significantly worse OS than those without a metastasis (median OS, 244 vs. 437 days, respectively, *p* < 0.0001; Figure 3(b)). As expected, we also found that patients with multiple metastases have significantly worse survival outcomes than those with no or a single metastasis at diagnosis (median OS, 175 vs. 351 days, respectively, *p* < 0.0001; Figure 3(c)) and, furthermore, that patients with three or more metastases at diagnosis have lower survival rates than those with at most two metastases (median OS, 183 vs. 344 days, respectively, *p* < 0.0027; Figure 3(d)). Lastly, patients with at least four metastases also exhibited worse OS than those with less than four metastases at diagnosis (median OS, 31.5 vs. 343 days, respectively, *p* < 0.0001; data not shown). However, given the extremely small number of events (two) in the group of patients with four or more metastases, this result should be interpreted with caution.

Importantly, a basic multivariate Cox's regression analysis (including standard clinicopathological parameters such as patients' age [as a continuous variable], gender, smoking status, number of metastases at diagnosis, and localization of the primary tumor) also showed that the number of metastases at diagnosis predicted the survival outcomes independent of other variables (hazard ratio [HR] 1.815, 95% CI 1.552–2.123, *p* < 0.001; Table 3). Besides, as expected, the patients' age also influenced the survival outcomes in the multivariate model (HR 1.031, 95% CI 1.016–1.047, *p* < 0.001). However, the other parameters, including tumor location, smoking status or gender, had no significant impact on survival. It is important to note, however, that performing a Schoenfeld residuals test on the above variables revealed that the covariate encoding the number of metastases at the time of diagnosis had a nonconstant hazard in time, thus the basic model should have been slightly adjusted. Thus, to resolve this violation of the proportional hazards' assumption, we included an interaction term between the time and the number of metastatic sites at diagnosis (Table S1). This modified model resulted in a borderline significant contribution of the interaction term (HR 0.999, 95% CI 0.998–1.000, *p* = 0.048), which means that the effect of the number of metastatic sites at diagnosis somewhat decreases with time. This is a reasonable outcome, as it can be assumed that once a relatively long time passes after initial diagnosis and additional metastases onset, the original number of metastases would lose its relevance. When correcting for this effect, the number of metastases at diagnosis had a hazard ratio of 2.496 (95% CI 1.976–3.153, *p* < 0.001). Other variables included in the model remained nonsignificant.

**TABLE 3:** Multivariate Cox regression model for clinicopathological variables influencing the OS

		OS
Age (continuous)		
	HR	1.031
	95% CI	(1.016–1.047)
	*p*	<0.001
Gender (male vs. female)		
	HR	1.061
	95% CI	(0.822–1.369)
	*p*	0.650
Localization (peripheral vs. central)		
	HR	0.891
	95% CI	(0.619–1.282)
	*p*	0.534
Smoking status (never vs. current/ex)		
	HR	0.850
	95% CI	(0.417–1.734)
	*p*	0.656
Number of metastases at diagnosis: 0/1/2/3/4		
	HR	1.815
	95% CI	(1.552–2.123)
	*p*	<0.001

*Note*: Concordance = 0.689. CI, confidence interval; OS, overall survival; HR, hazard ratio.

## DISCUSSION

Two‐thirds of SCLC patients present with metastatic disease at diagnosis and SCLC‐related deaths are mainly attributable to extrathoracic metastasis.^18^ Distant SCLC organ metastases can also cause corresponding symptoms which are associated with loss of functional independence and reduction in quality of life.^15^ Accordingly, understanding the metastatic patterns in advanced‐stage SCLC is crucial for clinical case management and might help in the development of individualized therapeutic strategies.^19^

The influence of primary tumor location on the site preference and timing of distant organ metastases has been studied in different solid tumors, including NSCLC, and colorectal‐ and pancreatic cancer.^9^, ^20^, ^21^, ^22^, ^23^ Our group previously found that centrally located lung adenocarcinomas give rise to bone metastases, and are associated with early metastatic spread and impaired OS compared to peripheral tumors.^9^ In case of colorectal cancer, right‐sided tumors are mostly associated with lung metastases, while left‐sided are associated with liver metastases.^20^, ^21^, ^22^ Yet, to our knowledge, our study is the first investigating the impact of primary SCLC location on organ specificity and timing of distant organ metastasis and, moreover, on patients' survival. As expected, and in line with previously published data,^24^ our results revealed that a considerable proportion of SCLCs were centrally located. Of note, because there are no standard definitions for central vs. peripheral lung tumors, in our study the exact localization of the primary tumor was defined based on its bronchoscopic visibility. Nevertheless, we found no significant association between primary tumor location and timing or organ preference of distant metastases. As for its prognostic relevance, although patients with centrally located tumors exhibited inferior median OS compared to those with peripheral tumors, this tendency does not appear to be statistically significant.

Next, in order to assess the clinical relevance of metastasis pattern in SCLC, we investigated the timing and co‐occurrence of organ metastases. With regards to the incidence of distant metastases, the most common metastatic sites were the liver, brain, bone, and adrenal glands, which is in line with the findings of others.^5^, ^10^, ^25^ Importantly, we also found that the onset of bone, brain, and pericardial metastases tended to be late, whereas the development of adrenal gland and liver metastases were usually early events during SCLC progression. Sequential organ‐specific colonization with short or long latency periods was observed in several tumor types, including NSCLC, and colorectal and breast cancer.^9^, ^26^, ^27^ To date, however, no sequence‐specific metastasis patterns have been reported in Caucasian SCLC patients. The timing of metastases is influenced by a series of stochastic events such as local invasion and intravasation, dissemination in the circulation, arrest at the distant site, extravasation, and invasion of the target tissue.^28^, ^29^ Because of its anatomical localization and vascular features, the liver is the most common site of distant metastasis in solid tumors.^28^ It is not surprising, therefore, that the occurrence of liver metastases tended to be an early event during SCLC progression. In contrast, the onset of brain metastases are usually late events because tumor cells need a significantly longer time to extravasate into the brain than into any other organ due to the morphological structure of the blood–brain barrier.^30^, ^31^ Interestingly, when analysing the co‐occurrence of metastases we found that bone metastases tended to appear together with liver metastases, while brain metastases occurred together with adrenal metastases. These results are supported by the findings of Wang et al. and Cai et al., who also found that liver and bone metastases were the most common combination of metastatic sites in Asian lung cancer patients.^10^, ^19^ Furthermore, similar conclusions were drawn in a recent autopsy study.^32^ The mechanisms underlying the co‐occurrence of different organ metastases in SCLC remains to be elucidated. However, it is suspected that in case of bone and liver metastases the similarity of the microvascular wall might play a key role.^28^ Accordingly, both in the bone and liver the vasculature is fenestrated and poses a lower physical barrier than in other organs.^27^, ^28^, ^33^ With regards to the co‐occurrence of brain and adrenal gland metastases, to our knowledge, to date, no similar data has been reported in lung cancer patients and the mechanisms that lie behind this observation are unknown. Accordingly, for bone‐metastatic SCLC patients, liver ultrasound and CT scan, and for those with adrenal gland metastases, prophylactic cranial irradiation (PCI) might be considered. Altogether, our results regarding the timing and co‐occurrence of organ metastases are of clinical importance, indicating the need for individualized treatment decisions and follow‐up strategies.

Distant metastases are a major factor for unfavourable prognosis in SCLC, therefore we also investigated the prognostic relevance of organ metastases. Our results revealed a significant decrease in OS with the increasing number of metastatic sites. In support of this, multivariate Cox regression analysis also revealed that the number of metastases was an independent predictor for worse OS. These results are consistent with the findings of others.^5^, ^34^, ^35^, ^36^ With regards to the impact of distinct metastatic sites on survival, we observed a clinically relevant decrease in OS in patients with brain metastasis at diagnosis, although these results were not statistically significant. In line with this, other researchers also revealed that patients with brain metastases tend to have poorer survival outcomes in SCLC.^37^, ^38^ Of note, the prognosis for patients with brain metastases is generally poor (irrespective of cancer type) mainly due to poor performance status, associated neurologic symptoms, and cerebral oedema.^39^, ^40^ Moreover, another common feature in most brain metastases is resistance to therapy, which is attributed to the poor penetration of therapeutics across the blood–brain barrier.^41^

The present study had certain limitations given by its retrospective nature. First, no information was available on the metastasis‐specific therapeutic approaches, including bisphosphonate therapy in case of bone metastases or WBRT in case of brain metastases. Second, due to the relatively long time period, diagnostic methods and treatment guidelines may have changed over the years which might also influence the prognosis. Third, no information was available on the utilization of PCI. However, PCI did not lead to increased OS in a recent phase III clinical trial.^42^ Fourth, the majority of included patients were evaluated by clinical and not by pathological TNM staging. Moreover, modern imaging methods like FDG PET‐CT or bone scintigraphy were not used as standard examination methods in the present cohort and most distant organ metastases were diagnosed by CT scan and MRI. Hence, we were not able to diagnose asymptomatic or micro‐metastases, which may have led to an underestimation of patients with certain types of metastases. Nevertheless, in line with clinical practice, most included patients were staged accurately at diagnosis and at recurrence. Fifth, our methodology dividing the primary tumors into central and peripheral lesions based on bronchoscopic visibility might also cause bias in some results. To date, however, there are no standard definitions for central vs. peripheral lung tumors.^9^ All in all, taking into account all the aforementioned potential study limitations, caution is needed when interpreting the results of the present study.

To conclude, our results are suggestive for particular site‐ and sequence‐specific metastasis patterns in patients with SCLC. We found that the onset of bone, brain, and pericardial metastases are late events, whereas adrenal gland and liver metastases occur earlier during tumor progression. Notably, several metastatic sites, such as bone and liver, and brain and adrenal gland, tended to co‐metastasize preferentially. As for their prognostic relevance, we found that the number of metastases at diagnosis is an independent predictor for worse survival outcomes. Furthermore, this is the first study investigating the impact of primary tumor location on the metastasis pattern in SCLC, yet no significant associations were observed with regards to the timing and organ preference of distant metastases when comparing central and peripheral primary tumors. Altogether, our findings might contribute to the development of new therapeutic approaches and might help in the development of individualized treatment decisions and follow‐up strategies in advanced‐stage SCLC patients.

## CONFLICT OF INTEREST

The authors declare no conflict of interest.

## Supporting information


**Table S1** Multivariate Cox regression model for clinicopathological variables influencing the overall survival (with an added interaction term)
