# Supplementary material for: The landscape of small cell lung cancer metastases: Organ specificity and timing
Source: Thorac Cancer. 2021 Feb 3;12(6):914–23. doi: 10.1111/1759-7714.13854 (PMC7952793; doi:10.1111/1759-7714.13854)
Supplement: Supplementary file 1 — Table S1 Multivariate Cox regression model for clinicopathological variables influencing the overall survival (with an added interaction term) [file TCA-12-914-s001.docx]

| **Supplementary Table 1. Multivariate Cox Regression model for clinicopathological variables influencing the OS (with an added interaction term)** | | |
| --- | --- | --- |
|  |  | **OS** |
| **Age (continuous)** |  |  |
|  | HR | **1.000** |
|  | 95% CI | (0.999-1.000) |
|  | *p* | **0.901** |
| **Gender (male vs. female)** |  |  |
|  | HR | **1.028** |
|  | 95% CI | (0.761-1.389) |
|  | *p* | **0.856** |
| **Localization (peripheral vs. central)** |  |  |
|  | HR | **0.999** |
|  | 95% CI | (0.655-1.523) |
|  | *p* | **0.995** |
| **Smoking status (never vs. current/ex)** |  |  |
|  | HR | **1.811** |
|  | 95% CI | (0.945-3.468) |
|  | *p* | **0.073** |
| **Number of metastases at diagnosis: 0/1/2/3/4** |  |  |
|  | HR | **2.496** |
|  | 95% CI | (1.976-3.153) |
|  | *p* | **<0.001** |
| **Interaction term:** Time: Number of metastases at diagnosis (0/1/2/3/4) | | |
|  | HR | **0.999** |
|  | 95% CI | (0.998-1.000) |
|  | *p* | **0.048** |
| OS: overall survival; HR, hazard ratio; CI, confidence interval; concordance = 0.709 | | |
